# Modeling the path to digital health intention: the mediating role of system expectation and health beliefs

**DOI:** 10.3389/fpubh.2025.1697273

**Published:** 2025-10-07

**Authors:** Bowen Pu, Kuoliang Huang

**Affiliations:** Department of Industrial Design, Design Academy, Sichuan Fine Arts Institute, Chongqing, China

**Keywords:** mobile health (mHealth), persuasive systems design, health belief model, user adherence, behavioral intention, population health, chronic disease prevention, PLS-SEM

## Abstract

**Introduction:**

While mobile health (mHealth) offers a seemingly scalable solution to the persistent challenge of chronic disease prevention, its real-world public health impact has arguably been blunted by a single, stubborn issue: low user adherence. The difficulty, in our view, stems from a tendency in the existing literature to treat technology and user psychology as separate domains. This creates what we call a theoretical “black box” between the features of a digital intervention and the behavioral outcomes it is meant to produce. Without a clearer picture of what happens inside this box, efforts to create truly data-driven and effective population-level interventions remain somewhat handicapped.

**Methods:**

A self-administered online survey using Wenjuanxing (wjx.cn) was undertaken in a cross-sectional design. Chinese adults (≥18 years) with pre-existing exposure to or intention to use digital health were the target population; a non-probability, voluntary sampling frame yielded 620 usable surveys after screening for quality. The psychometrics were tested, and screening of common-method bias (full-collinearity VIF) preceded testing of structural paths and serial mediation from persuasive features (functional/experiential) to system expectations and through to health beliefs to intention using PLS-SEM.

**Results:**

The data showed that Persuasive Experiential Support (PES) was a key antecedent for Integrated System Expectation (ISE), which in turn stood out as the strongest predictor of Persuasive Health Belief (PHB). Interestingly, we also uncovered a substantial measurement overlap between our PHB construct and Behavioral Intention (BI)—a finding that points toward a potential “belief-intention fusion” process in these kinds of highly persuasive digital environments.

**Conclusion:**

Taken together, these results seem to advocate for what might be called an “experience-first, function-as-assist” design philosophy for mHealth interventions targeting chronic disease at scale. In other words, prioritizing an engaging user experience looks to be a critical precondition for building the system trust needed to actually foster health beliefs and drive intentions. Perhaps more importantly, our unexpected finding regarding belief-intention fusion opens up a new, testable research agenda—one that explores how real-time digital interactions might be fundamentally reshaping the cognitive pathways of decision-making. This is a crucial question for the next generation of AI-driven, population-level health promotion tools.

## 1 Introduction

There is an understandable excitement around digital health technologies, particularly mobile health (mHealth) applications, which seem to hold enormous potential for tackling chronic diseases at a population scale (1, 2). The reality on the ground, however, has proven to be a bit more complicated. A stubborn gap persists between this technological promise and its actual public health impact, a gap largely attributable to one persistent factor: low user adherence (3–5). This is not a minor issue; it’s a critical bottleneck often called the “adherence crisis,” which consistently prevents promising digital interventions from achieving any kind of sustainable, population-level improvement (6, 7). Nowhere is this challenge more evident than in China’s burgeoning mHealth market. While high mobile penetration has fueled impressive initial adoption (8, 9), nowhere is this challenge more evident than in China’s burgeoning mHealth market. While high mobile penetration has fueled impressive initial adoption (10–12). All of this leads to what we see as a crucial question for the field: How, then, can we systematically design the features of these technologies to more effectively engage users by leveraging psychological mechanisms that we know are valid?

Part of the difficulty in answering the question we just posed seems to lie in how the literature has traditionally been structured. You have, on one side, a body of work from computer science and HCI, like the Persuasive Systems Design (PSD) model, which is quite good at cataloging design features that can prompt behavioral change (13). But the focus there is almost entirely on the technological input, with the psychological “how” and “why” often left unexamined. Then, on the other side, you have these foundational theories from health psychology, such as the Health Belief Model (HBM). They give us a rich understanding of cognitive drivers like risk perception, but they tend to treat technology as if it were just a neutral delivery system—a questionable assumption in today’s interactive digital world (14, 15). What we are left with is this disciplinary split, this theoretical gap. It’s a “black box” that is not just an academic curiosity; it’s a very real barrier that complicates our efforts to design digital public health interventions that are both effective and truly scalable (16, 17).

This research aims to bridge that divide by synthesizing PSD and HBM into a single, process-oriented framework. We propose and empirically test a multi-stage serial mediation model that maps the full trajectory from persuasive technology design to health behavior intention. Our model introduces and validates the construct of Persuasive Health Belief (PHB), which captures the unique, technology-facilitated belief system that emerges when users interact with a persuasive digital platform. This approach allows us to answer central questions with direct implications for digital public health:

*RQ1:* Which persuasive design features—functional versus experiential—are most critical for shaping users’ initial perceptions and trust?

*RQ2:* How do these initial perceptions consolidate into technology-mediated health beliefs?

*RQ3:* And finally, within these digitally mediated environments, does the journey from belief to intention follow a traditional linear path, or does it transform into a more dynamic, fused process?

By empirically examining the psychological sequence of “expectation formation → belief consolidation → action empowerment,” this work offers three central contributions to the nexus of technology and public health. First, it provides a testable and actionable framework that demonstrates how specific design features can be leveraged to drive health behavior intentions, offering a roadmap for evidence-based intervention design. Second, it introduces the novel PHB construct, providing a new conceptual tool for understanding and measuring belief systems within technologically mediated spaces. Third, by uncovering and grappling with a critical statistical anomaly, it proposes a “belief-intention fusion” dynamic that challenges classical theories and may unlock more potent strategies for just-in-time digital interventions. Ultimately, our work seeks to inform a new, experience-centric design paradigm for next-generation digital health systems, providing theoretical and practical support for tackling the enduring adherence crisis in population health.

## 2 Research review and hypothesis development

To get to our hypotheses, it seems necessary to first grapple with the explanatory power, and indeed the limitations, of the two major frameworks that dominate this space: PSD and the HBM. This next section is really about clarifying the distinct roles that design features, on the one hand, and cognitive beliefs on the other, play in shaping health behavior. In doing so, we hope to shed more light on that theoretical “black box” we mentioned, the one that currently sits between technological input and behavioral output. We think that by properly identifying this gap, we can then establish a more integrative path forward for our hypotheses—a path designed to trace the full psychological journey from a system’s persuasive features to a user’s eventual behavioral intention.

### 2.1 Bridging the theoretical divide: why PSD and HBM must be integrated in digital health

Within digital public health research, it’s probably fair to say that PSD and HBM represent the two main theoretical pillars used for thinking about health behavior change. Coming out of the human-computer interaction world, the PSD model gives us a fairly systematic way to design digital systems that can nudge behavior, offering what is essentially a practical, design-focused roadmap (18). The Health Belief Model, a cornerstone of health psychology, comes at the problem from a different angle entirely. HBM is really all about the cognitive antecedents of our health-related choices. It posits that an individual’s likelihood of adopting a health behavior is governed by a set of core beliefs—things like their perceived susceptibility to a health threat, the severity of that threat, the pros and cons of taking action, and, crucially, their own self-efficacy (14).

**Figure 1:**
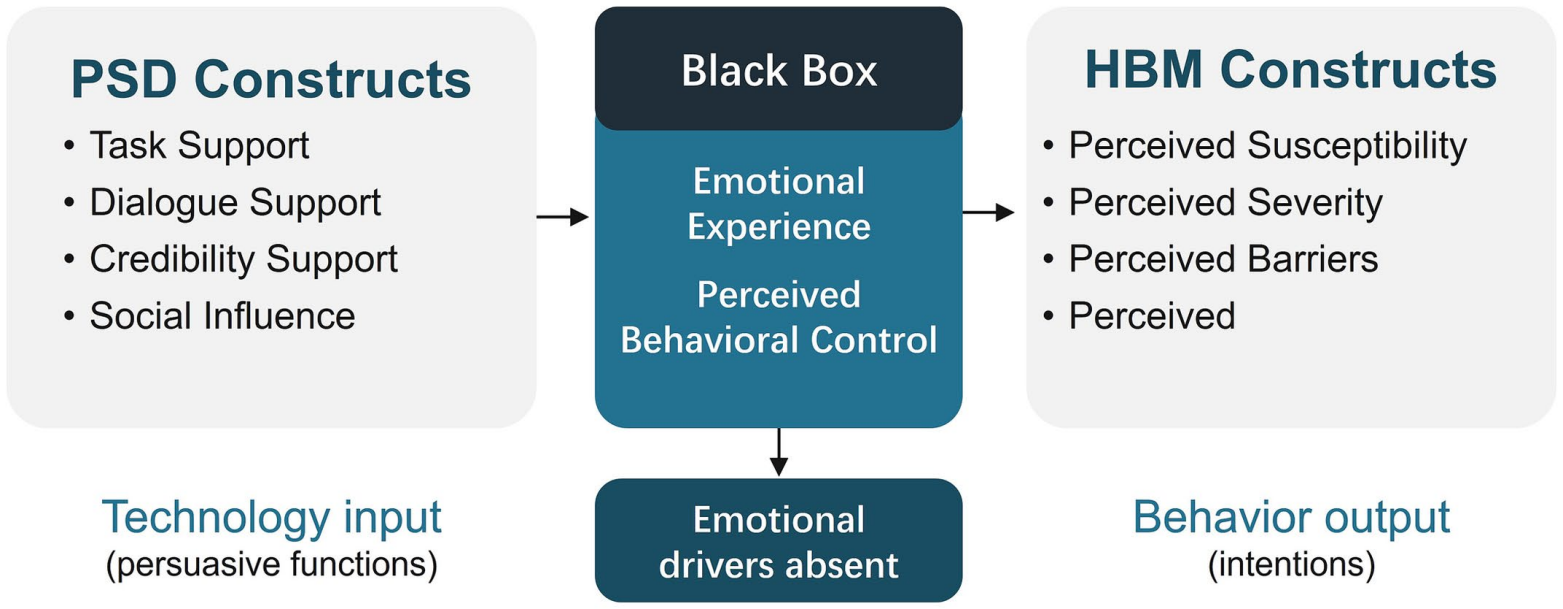
The theoretical gap in integrating PSD and HBM in eHealth research.

While both models have demonstrated considerable utility, their parallel development has created a theoretical bifurcation, pitting technologically deterministic explanations against purely cognitive ones with little cross-fertilization (13). PSD adeptly answers the “how” of persuasive design but often fails to explain the “why” of its behavioral effects from a psychological standpoint. HBM, in contrast, excels at explaining the “why” of human motivation but frequently treats the technology delivering the health message as a passive information conduit rather than an active persuasive agent—a particularly problematic assumption in the dynamic, interactive context of mHealth (19). As depicted in Figure 1, this disciplinary silo has left a critical “black box” unexamined: How, precisely, do the designed features of a persuasive system translate into the foundational beliefs that drive health behavior?

This theoretical gap has profound practical consequences for population health. It hinders the development of a unified theory of digital health behavior change and risks generating fragmented or incomplete design guidance for real-world interventions (20). To overcome this, we argue for an interdisciplinary synthesis of PSD and HBM. Such a framework is essential to illuminate the entire causal chain—from technology input to psychological mediation to behavioral intention—and to provide a more complete, evidence-based roadmap for designing digital health interventions that are not only technologically sound but also psychologically resonant and effective at scale.

### 2.2 Experience and agency: emerging psychological drivers in digital health behavior

If you look at traditional health behavior models, whether it’s the HBM or the Theory of Planned Behavior (TPB), they tend to be grounded in some fairly rationalist assumptions. The emphasis is almost always on deliberate, cognitive evaluation as the main driver of our decisions (21). The issue, of course, is that in the highly interactive and feedback-rich digital world of mHealth, things are rarely so straightforward. Affective and experiential cues often seem to play a much more decisive role in guiding what users actually do. This is a dimension that, for the most part, gets neglected by these conventional theories. So, to try and get a fuller picture, our own model makes a point of incorporating two what we believe are crucial psychological drivers: Persuasive Experiential Support (PES), to represent the affective pathway, and Perceived Behavioral Control (PBC), to capture the user’s sense of agency.

Let us take PES first. In contrast to the more functionally oriented design features you might see—what we call Persuasive Functional Support, or PFS—PES is all about the emotionally resonant elements. We’re talking about things like the visual aesthetics of the app, gamification, social comparison features, and affirmative feedback. The whole point of these features is to create user experiences that are intrinsically engaging and positive (22–24). And these kinds of experiences appear to be critical. They seem to do more than just foster initial trust and positive expectations toward the system—a construct we have termed Integrated System Expectation (ISE). They also seem to boost a user’s self-efficacy simply by placing the target behavior in a context that feels emotionally supportive and reinforcing (25, 26).

Within our framework, PBC—drawn from TPB—serves as a vital psychological bridge linking system-level support to the user’s sense of capability. It reflects an individual’s confidence and perceived agency in their ability to enact a specific health behavior, a perception shaped by both internal competencies and the enabling affordances of the mHealth platform (27). Whether fostered by the structured guidance of PFS or the affective reinforcement of PES, strong persuasive design can significantly enhance PBC, thereby increasing the likelihood that a user’s intentions will translate into concrete action (28). By integrating both emotional engagement (PES) and perceived capability (PBC), this study advances a more holistic and context-sensitive framework that better reflects the multifaceted psychological underpinnings of intention formation in modern, interactive digital health environments.

### 2.3 Hypothesis development: a multi-stage serial mediation framework

**Figure 2:**
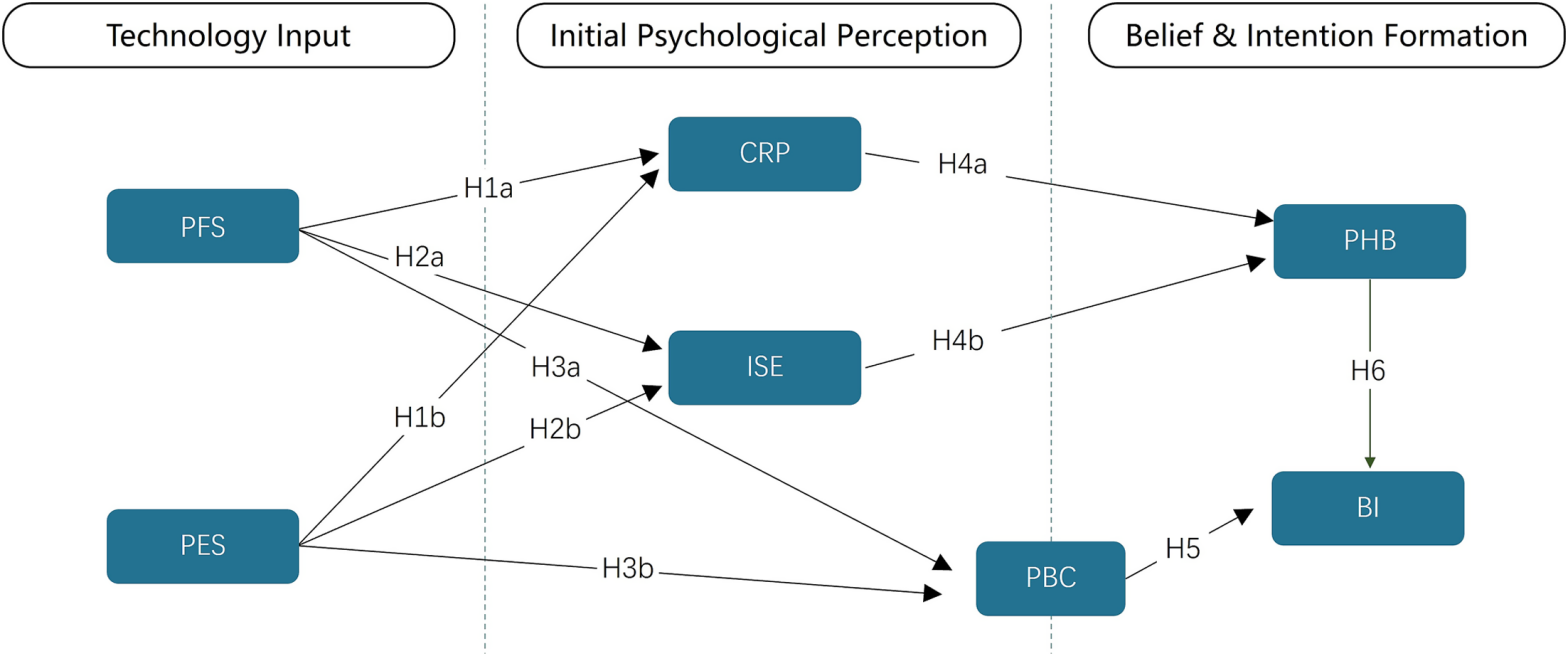
Integrated PSD-HBM theoretical model and research hypotheses. PFS, Persuasive Functional Support; PES, Persuasive Experiential Support; CRP, Comprehensive Risk Perception; ISE, Integrated System Expectation; PHB, Persuasive Health Belief; BI, Behavioral intention.

To empirically dissect the theoretical “black box” connecting technological input, psychological processing, and behavioral output, this study proposes an integrative, multi-stage serial mediation model (see Figure 2). The model posits a clear psychological pathway: persuasive technological features (the input layer) do not directly determine user behavior. Instead, they first trigger a set of initial psychological perceptions about the system and the self (the first mediator layer), which in turn foster the consolidation of more stable, technology-contextualized health beliefs (the second mediator layer). It is these beliefs, in concert with perceived behavioral control, that ultimately shape a user’s behavioral intentions (the output layer) (29, 30).

#### 2.3.1 Phase one—how persuasive features shape early psychological perceptions (RQ1)

In the initial phase of user engagement, PFS and PES are theorized to sculpt early perceptions across cognition, emotion, and control. PFS—goal-oriented utilities such as self-monitoring, reminders, and personalized guidance—helps users access and interpret health information, scaffolds workflows, and increases instrumental efficacy; thus, it strengthens CRP, consolidates ISE (via trust in system capability), and elevates PBC (18). PES—aesthetics, praise, rewards, social feedback—creates intrinsically engaging experiences that buffer defensive reactions to risk, heighten credibility and positive affect, and raise competence feelings; accordingly, PES supports CRP/ISE/PBC (31). We therefore test: H1. PFS and PES → CRP; H2. PFS and PES → ISE; H3. PFS and PES → PBC (18, 31).

Hypotheses as stated in the manuscript:

*H1:* Both (a) PFS and (b) PES positively predict CRP.

*H2:* Both (a) PFS and (b) PES positively predict ISE.

*H3:* Both (a) PFS and (b) PES positively predict PBC.

#### 2.3.2 Phase two—from perception to belief: constructing technology-mediated health beliefs (RQ2)

**Figure 3:**
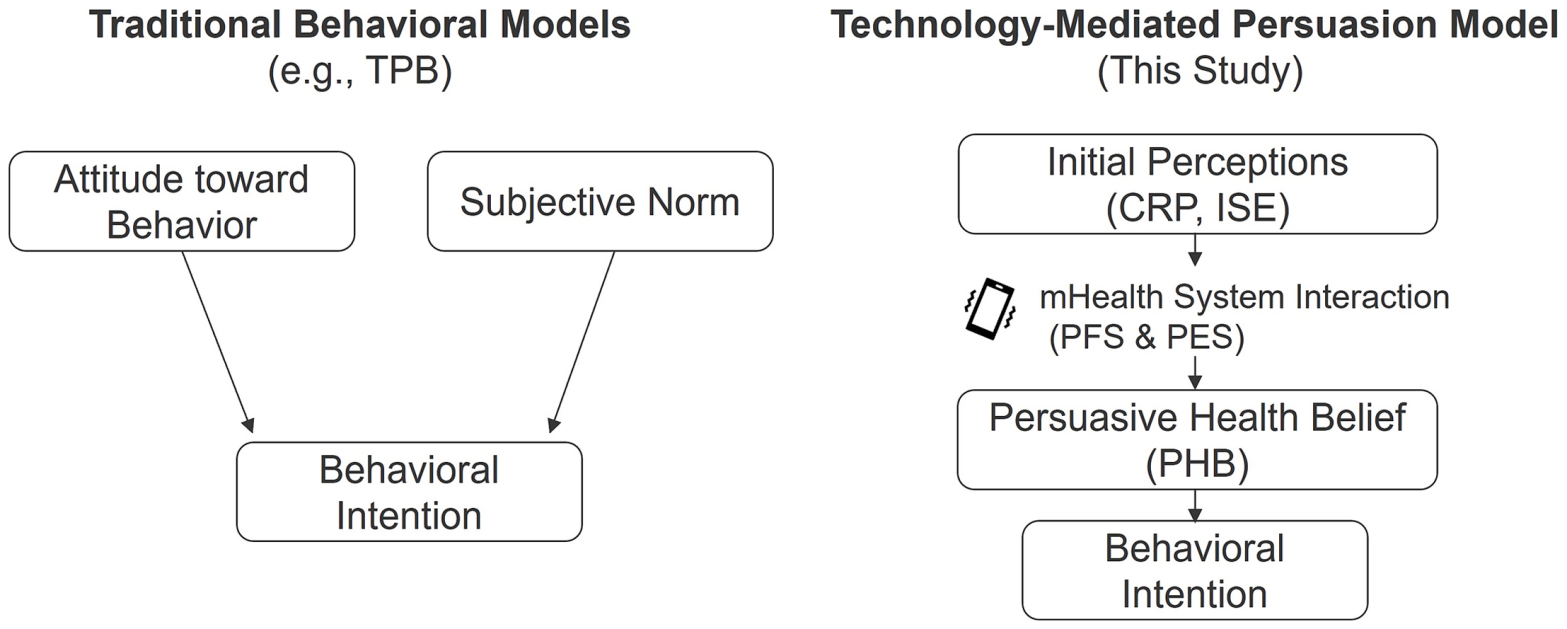
Conceptual distinction of PHB.

Following initial appraisal, perceptions consolidate into PHB—a technology-mediated, hybrid belief system that fuses evaluation of the target behavior with endorsement of the mHealth system as a legitimate persuasive agent. As shown in Figure 3, belief in the target health behavior and trust in the persuasive mHealth system are co-constructed, distinguishing PHB from conventional, system-agnostic attitudes. PHB is more context-bound and dynamic than TPB “attitude,” because it is co-constructed through ongoing human–system interaction (21). Within this pathway, CRP provides a cognitive anchor, whereas ISE catalyzes belief consolidation by extending trust from the system to its recommended behaviors (risk and trust routes). Evidence shows that heightening risk appraisals can shift intentions/behaviors, underscoring CRP’s role; experiential/credibility cues shape perceived usefulness and credibility in HCI, supporting ISE’s role (31, 32). Hence,

*H4:* (a) CRP and (b) ISE each positively predict PHB.

#### 2.3.3 Phase three—joint drivers of intention: health belief and control (RQ3)

Within the TPB, behavioral intention is the most proximal antecedent of action and is shaped jointly by belief structures and perceived behavioral control (21). Large-scale meta-analyses confirm that TPB constructs predict health behaviors prospectively, while also documenting a persistent intention–behavior gap that highlights the importance of control-related appraisals and action-enabling design (33–35). In digital, feedback-rich mHealth settings—where AI-personalized prompts and continuous interaction can rapidly strengthen (or erode) motivation—technology-mediated beliefs and perceived capability are therefore expected to co-determine intention at scale (27, 36).

Consistent with our framework, PHB captures a technology-mediated belief system that fuses evaluations of the target health behavior with trust in the mHealth system as an active persuasive agent. PBC indexes users’ capability and agency to enact the behavior (21). Together, PHB and PBC provide complementary motivational and volitional bases for intention: PHB energizes commitment to the recommended action, while PBC supports feasibility judgments and plan enactment. This dual-driver view aligns with evidence that increasing risk appraisals and control beliefs can shift intentions and downstream actions, yet those intentions translate imperfectly into behavior without sufficient control and action support (34, 35).

*H5:* PHB positively predicts Behavioral Intention (BI).

*H6:* PBC positively predicts BI.

## 3 Materials and methods

### 3.1 Research design and ethical considerations

For this study, we decided on a quantitative, cross-sectional survey design. Essentially, this allowed us to capture a snapshot in time of the complex relationships between the technology inputs, the psychological mediators we have proposed, and the eventual behavioral outcomes (37). This kind of design seemed particularly well-suited for our primary goal here, which was really about testing and validating the theoretical model itself.

In conducting this research, we took care to adhere to the ethical principles outlined in the Declaration of Helsinki. Naturally, all procedures were formally reviewed and approved by the Ethics Review Committee of the School of Design at Sichuan Fine Arts Institute, and we made sure that every participant was well-informed before taking part. This involved providing a detailed electronic information sheet and securing digital informed consent to ensure participation was wholly voluntary. All data were subsequently collected and stored anonymously to protect participant privacy and confidentiality.

### 3.2 Participants and sampling procedure

The target population for this study comprised Chinese adults with prior experience or familiarity with digital health technologies. Eligibility criteria required participants to be 18 years or older and to affirm at least one of the following: (1) previous use of health-related technologies (e.g., wellness apps, smart wearables); (2) a functional understanding of digital health concepts; or (3) a stated willingness to use technology for personal or family health management. These criteria ensured that respondents possessed the necessary contextual knowledge to provide meaningful and valid responses to the survey items.

Data were collected via “Wenjuanxing”,^1^ a leading online survey platform in mainland China, using a non-probability voluntary response sampling method. While non-probability sampling limits population-level generalizability, it is a widely accepted and practical approach for exploratory, theory-driven research in health information systems and behavioral science, where the primary objective is to test theoretical relationships between variables rather than to generate precise population estimates (38, 39). Specifically, it implies here that though the results are strong in testing the theory-based relations among the construct variables, their direct generalizability to the overall Chinese adult population might be limited. This limitation points to the necessity of subsequent studies by using probability sampling procedures to establish these results in larger and more representative populations. To enhance data quality, we implemented several control measures: initial screening questions confirmed eligibility, and automated checks were used to detect and exclude responses with excessively short completion times (under 80 s) or invalid patterns (e.g., straight-lining), thereby minimizing low-quality or inattentive submissions.

### 3.3 Measurement instrument

A structured questionnaire was developed to operationalize all key constructs in the theoretical model. All measurement items were adapted from validated scales established in prior research and were carefully localized to fit the persuasive mHealth context, ensuring both content validity and contextual relevance (see Table 1). A 7-point Likert scale (1 = “strongly disagree” to 7 = “strongly agree”) was used for all responses.

**Table 1:** Model constructs, operational definitions, and measurement sources.

Construct	Operational definition	Source of scale
Persuasive Functional Support (PFS)	Users’ perceptions of the system’s functions that facilitate behavior change.	DeLone and McLean (70)
Persuasive Experiential Support (PES)	Users’ subjective experiences during system use.	Bartneck et al. (71)
Comprehensive Risk Perception (CRP)	Users’ perceived overall threat regarding a specific health issue.	Venkatesh et al. (51)
Integrated System Expectation (ISE)	Users’ overall judgment of the effectiveness and outcome expectations of the mHealth system.	Oinas-Kukkonen (72) and Ajzen (73)
Persuasive Health Belief (PHB)	A hybrid belief system deeply embedded in technology that dynamically integrates users’ cognitive evaluations of health behaviors (e.g., valence, efficacy) with sustained assessments of mHealth systems (e.g., system credibility, content trust).	
Perceived Behavioral Control (PBC)	Users’ confidence and sense of control over performing the health behavior.	Ajzen (73)
Behavioral Intention (BI)	Users’ intention or tendency to perform a specific health behavior in the future.	Ajzen (21); Fishbein and Ajzen (29)

The instrument creation embedded a comprehensive, multi-phased validation procedure to determine the instrument’s psychometric soundness. For the creation of robust content validity, an adapted two-round Delphi method was employed with a five-member group of domain experts in health behavior and human-computer interaction (40). This formal iterative process facilitated attaining expert consensus on the relevance, clarity, and theory coherence of the measurement items. The Delphi study protocol in full is presented in Appendix A. The exercise concluded once agreement was reached so that the finalized items possessed robust semantic clarity and cultural suitability and conformed to the theory framework (41). As a supplementary aide to respondent comprehension, the questionnaire comprised annotated diagrams of representative mHealth apps in the Chinese market. An important methodological consideration was the potential concept overlap between BI and PHB. Accordingly, we pre-registered to undertake strong statistical procedures—in short, the Heterotrait-Monotrait (HTMT) ratio and exploratory factor analysis (EFA)—to test their distinctiveness after recording. The complete questionnaire is presented in Appendix B.

### 3.4 Data analysis strategy

Data were analyzed using Smart PLS 4.0 with Partial Least Squares Structural Equation Modeling (PLS-SEM) as the primary statistical method. This approach was chosen for three reasons. First, PLS-SEM is highly effective for testing complex theoretical models with multiple causal pathways, making it ideal for our multi-stage mediation framework. Second, as a variance-based, non-parametric technique, it is robust to non-normal data distributions and performs reliably with medium to large sample sizes. Third, its emphasis on prediction aligns well with the study’s objective of developing a model with practical explanatory power for digital health interventions (42, 43).

The analysis followed a structured two-stage approach:

- *Measurement model evaluation:* This stage confirmed the instrument’s psychometric properties. We assessed (a) indicator reliability (factor loadings), (b) internal consistency (composite reliability, CR), (c) convergent validity (average variance extracted, AVE), and (d) discriminant validity using both the HTMT criterion and the Fornell-Larcker benchmark.
- *Structural model assessment:* After validating the measurement model, we tested the hypothesized relationships by examining: (a) path coefficient significance (*β*) via bootstrapping (5,000 resamples), (b) model explanatory power (*R*^2^), (c) predictive relevance (Stone-Geisser’s Q^2^), and (d) effect sizes (f^2^) for each path.

Before the main analysis, we formally tested for common method bias (CMB) using the full collinearity variance inflation factor (VIF) method. All VIFs were below the conservative threshold of 3.3, indicating that CMB was not a significant concern in this dataset (44).

## 4 Results

This section details the empirical findings from our data analysis (for raw data, see Appendix C). The results are presented in a structured, two-stage process: first, an evaluation of the measurement model to establish its reliability and validity, followed by an assessment of the structural model to test the proposed hypotheses (42).

### 4.1 Sample demographics and data quality

We started with an initial pool of 846 returned questionnaires, but we first ran these through a stringent screening process to ensure data quality. After filtering out responses that were completed too quickly (under 80 s) or showed invalid patterns like straight-lining, we were left with a final, valid dataset of 620 responses—an effective response rate of 73.3%. A quick *a priori* power analysis confirmed what we had hoped: that this sample size is more than adequate for detecting medium effect sizes within a PLS-SEM framework, giving us a solid foundation for the hypothesis tests that followed (42).

Looking at the demographic profile, the sample seems to mirror the typical user base for digital health tech in China quite well (45). Women made up a slight majority at 58.1%, and the 18–44 age bracket was the largest cohort (72.1%), which makes sense as this is the core market for health technology. It’s also worth noting, though, that a good portion of our sample (25.5%) was 45 or older, and we had a wide mix of usage frequencies from daily to infrequent users. This diversity should hopefully enhance the generalizability of our findings a bit.

### 4.2 Measurement model evaluation

The reflective measurement model was rigorously assessed to confirm its psychometric properties before proceeding to hypothesis testing.

#### 4.2.1 Reliability and convergent validity

As detailed in Tables 2, 3, the measurement model demonstrated excellent reliability and convergent validity. All standardized factor loadings (0.655 to 0.880) were statistically significant, confirming strong indicator reliability. Furthermore, all constructs exceeded established thresholds for internal consistency, with CR values ranging from 0.847 to 0.897 (benchmark > 0.70). Convergent validity was also robustly supported, as the AVE for each construct ranged from 0.544 to 0.774, well above the 0.50 benchmark (42, 46).

**Table 2:** Indicator load and 95% confidence intervals.

Construct	Item	Loading	CI_2.5%	CI_97.5%
PFS	PFS1	0.655	0.569	0.73
PFS	PFS2	0.772	0.699	0.818
PFS	PFS3	0.813	0.78	0.845
PFS	PFS4	0.752	0.688	0.806
PFS	PFS5	0.818	0.774	0.855
PFS	PFS6	0.801	0.751	0.842
PES	PES1	0.703	0.641	0.752
PES	PES2	0.813	0.769	0.848
PES	PES3	0.793	0.747	0.829
PES	PES4	0.777	0.719	0.819
PES	PES5	0.663	0.564	0.74
CRP	CRP1	0.695	0.619	0.759
CRP	CRP2	0.723	0.651	0.779
CRP	CRP3	0.813	0.775	0.848
CRP	CRP4	0.739	0.685	0.789
CRP	CRP5	0.648	0.574	0.717
ISE	ISE1	0.781	0.713	0.834
ISE	ISE2	0.773	0.711	0.819
ISE	ISE3	0.791	0.741	0.831
ISE	ISE4	0.756	0.694	0.795
ISE	ISE5	0.806	0.753	0.842
PHB	PHB1	0.742	0.679	0.787
PHB	PHB2	0.76	0.713	0.798
PHB	PHB3	0.709	0.628	0.771
PHB	PHB4	0.817	0.775	0.851
PHB	PHB5	0.651	0.581	0.707
PHB	PHB6	0.736	0.658	0.789
PBC	PBC1	0.829	0.783	0.868
PBC	PBC2	0.851	0.814	0.883
PBC	PBC3	0.81	0.751	0.857
BI	BI1	0.88	0.843	0.912
BI	BI2	0.88	0.836	0.912

**Table 3:** Construct-level internal consistency and convergent validity (CR and AVE).

Construct	k	AVE	CR	Min_Loading	Max_Loading	Min_CI_2.5%
BI	2	0.774	0.873	0.88	0.88	0.836
CRP	5	0.527	0.847	0.648	0.813	0.574
ISE	5	0.611	0.887	0.756	0.806	0.694
PBC	3	0.689	0.869	0.81	0.851	0.751
PES	5	0.565	0.866	0.663	0.813	0.564
PFS	6	0.594	0.897	0.655	0.818	0.569
PHB	6	0.544	0.877	0.651	0.817	0.581

#### 4.2.2 Discriminant validity and the emergence of a key empirical anomaly

**Figure 4:**
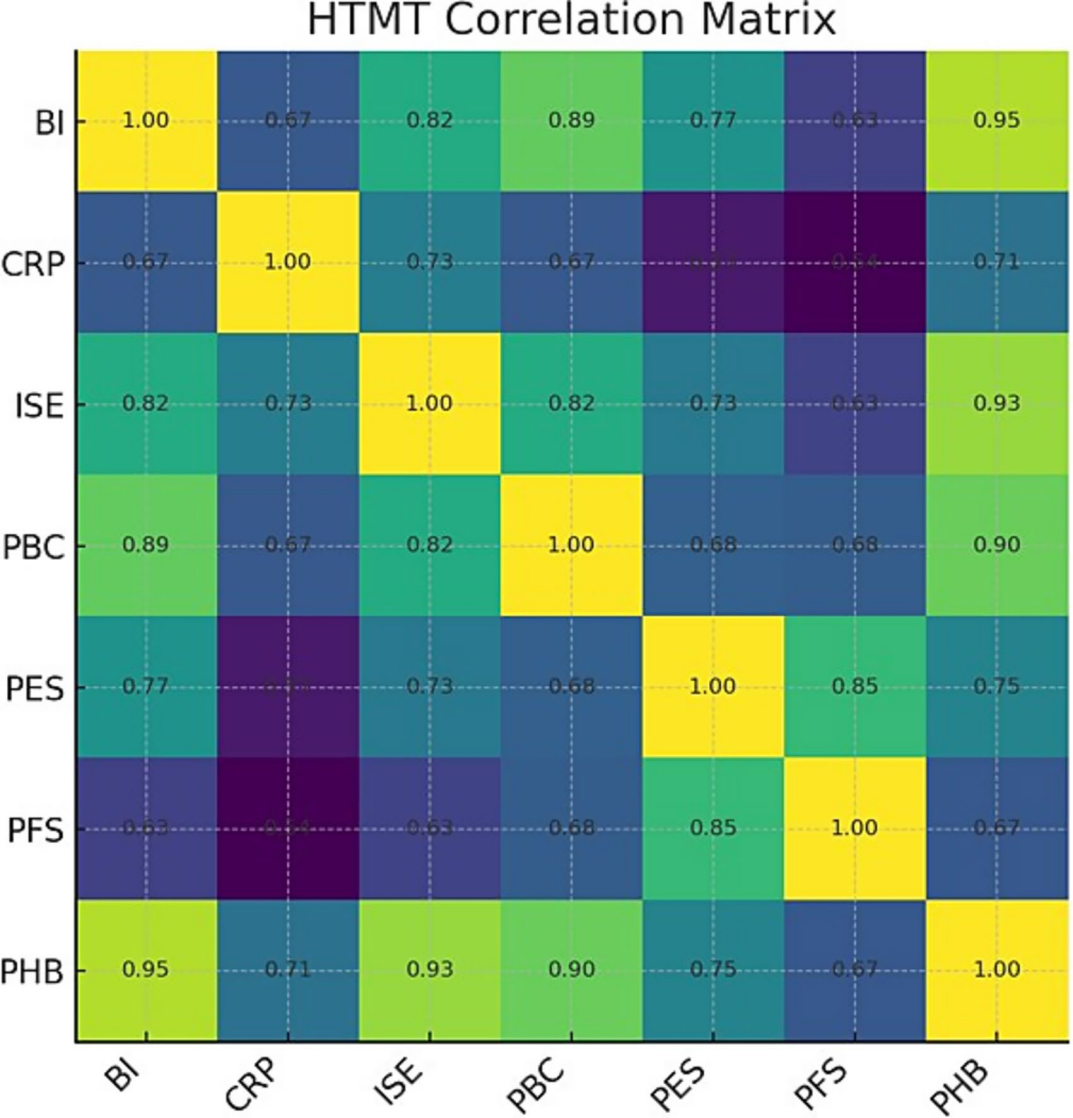
HTMT correlation matrix. Values on the off-diagonal are HTMT estimates; diagonal fixed to 1.00.

We assessed discriminant validity primarily using the HTMT ratio. As shown in the correlation matrix (Figure 4), most HTMT values were well below the conservative threshold of 0.85, indicating that the constructs were empirically distinct (47).

However, this analysis revealed a critical empirical anomaly: the HTMT value between our two central outcome constructs, PHB and BI, was 0.947. The 95% confidence interval for this value [0.900, 1.013] contained 1 (see Appendix D), suggesting a profound lack of discriminant validity and indicating that the two constructs were not empirically distinguishable in our sample.

**Figure 5:**
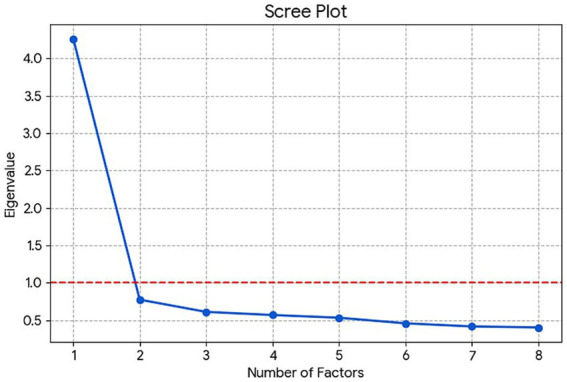
Scree plot for the combined PHB and BI Items.

Rather than dismissing this as measurement error, we treated it as a significant finding warranting deeper investigation. We conducted a follow-up Exploratory Factor Analysis (EFA) on the eight items corresponding to PHB and BI. The results strongly supported a unidimensional structure. The scree plot (Figure 5) revealed a distinct “elbow” after the first factor, which had a substantially larger eigenvalue than all subsequent factors, providing clear visual evidence for a single-factor solution (48).

Furthermore, a comparison of the one-factor and two-factor solutions (see Table 4) confirmed this conclusion. In the single-factor solution, all eight items loaded strongly (> 0.55) onto one cohesive factor. In contrast, the forced two-factor solution produced a theoretically incoherent structure with severe cross-loadings, failing to separate the BI and PHB items into distinct, meaningful factors.

**Table 4:** Exploratory factor analysis loadings for PHB and BI Items.

Item	One-factor solution	Two-factor rotated (F1)	Two-factor rotated (F2)
PHB1	0.685	**0.659**	0.240
PHB2	0.703	**0.546**	**0.449**
PHB3	0.639	**0.541**	0.338
PHB4	0.753	**0.617**	**0.429**
PHB5	0.558	0.215	**0.768**
PHB6	0.683	**0.663**	0.234
BI1	0.754	**0.644**	0.386
BI2	0.673	**0.650**	0.234

Loadings > 0.32 are considered significant. Bold indicates a theoretically messy or cross-loading structure in the two-factor solution.

Taken together, these results (high HTMT and a unidimensional EFA solution) provide robust evidence of a significant empirical overlap between belief and intention in this digitally mediated context (47, 48). Rather than interpreting this as a product of measurement, we interpret this anomaly itself as an underlying substantive outcome. It suggests an associative psychological “fusion” of intention and belief in highly persuasive online contexts such that the cognitive process of developing strong belief in a behavioral health outcome is empirically distinguishable in no way from developing the intention to adopt it. This interpretation elevates the outcome from a problem of statistical measurement to an underlying theoretical conclusion of this study and is discussed in depth within the Discussion. With this important caveat, the overall measurement model was deemed adequate for structural analysis.

### 4.3 Structural model assessment

After confirming the measurement model’s properties, we proceeded to test the theoretical hypotheses. A preliminary check for multicollinearity among endogenous constructs revealed that all Variance Inflation Factor (VIF) values were between 1.42 and 2.17, well below the conservative threshold of 3.3, indicating that multicollinearity was not a concern (44).

#### 4.3.1 Path analysis and hypothesis testing

**Figure 6:**
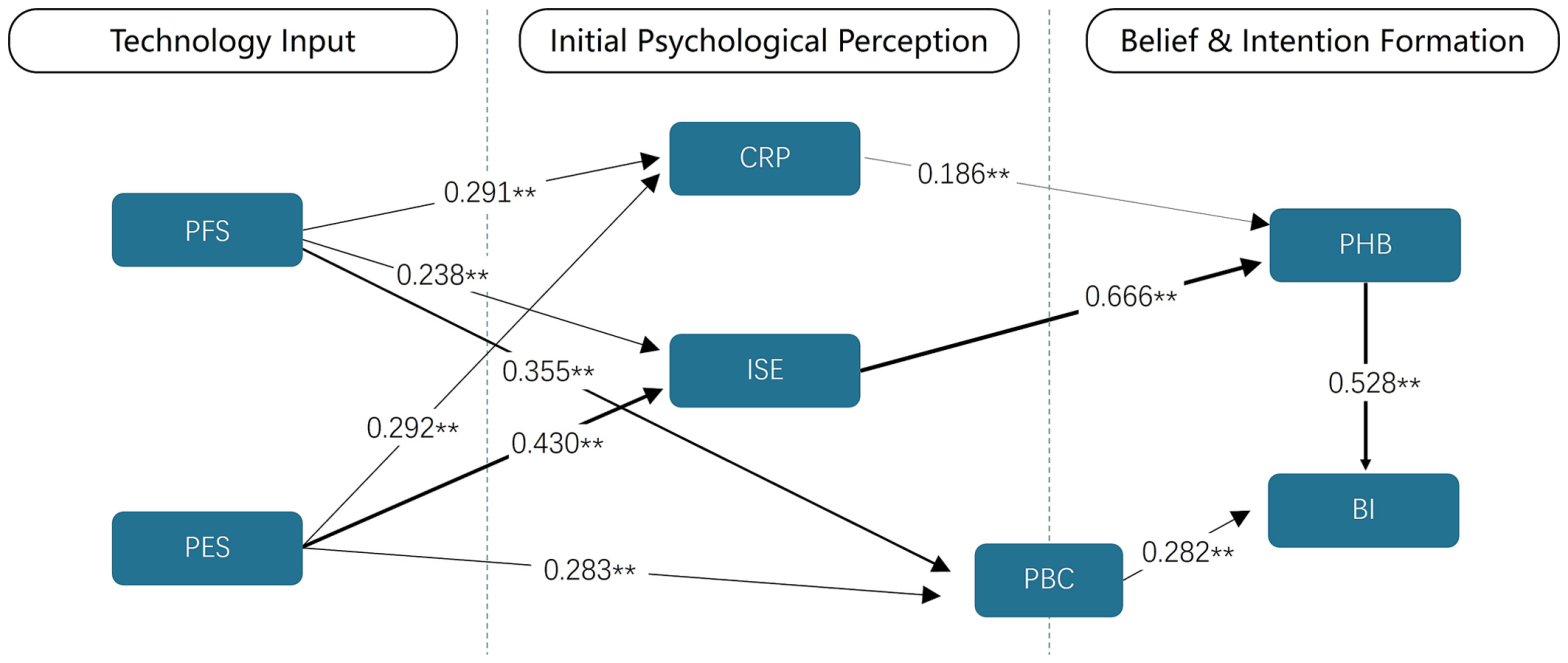
Results of the structural model path analysis. The figure clearly shows the multi-stage chain relationship from technical characteristics (PFS, PES) → initial psychological perception (CRP, ISE, PBC) → health beliefs (PHB) → behavioral intention (BI). The numbers on the arrows represent path coefficients (standardized *β* values), indicating the magnitude of the direct effects between variables. * *p* < 0.05; ** *p* < 0.01; Line thickness indicates path strength.

The significance of the hypothesized paths was evaluated using a bias-corrected bootstrapping procedure with 5,000 resamples. The results, summarized in Figure 6 and Table 5, provide strong support for our integrated model.

**Table 5:** Hypothesis test results.

Hypothesis	Structural path	*β* (Std.)	*t*	95% CI	Decision
H1a	PFS → CRP	0.291 (0.059)	4.941**	(0.169, 0.402)	Supported
H1b	PES → CRP	0.292 (0.057)	5.153**	(0.174, 0.398)	Supported
H2a	PFS → ISE	0.238 (0.055)	4.313**	(0.126, 0.342)	Supported
H2b	PES → ISE	0.430 (0.057)	7.567**	(0.307, 0.535)	Supported
H3a	PFS → PBC	0.355 (0.055)	6.461**	(0.242, 0.458)	Supported
H3b	PES → PBC	0.283 (0.057)	4.944**	(0.167, 0.390)	Supported
H4a	CRP → PHB	0.186 (0.037)	5.039**	(0.114, 0.259)	Supported
H4b	ISE → PHB	0.666 (0.034)	19.815**	(0.596, 0.726)	Supported
H5	PHB → BI	0.528 (0.055)	9.533**	(0.419, 0.638)	Supported
H6	PBC → BI	0.282 (0.062)	4.534**	(0.152, 0.400)	Supported

*p < 0.05, **p < 0.01; the number of Bootstrap samples for all paths is 5,000, and the confidence interval is the 95% CI after deviation correction.

The findings are organized according to the three phases of our proposed psychological pathway, corresponding to our research questions.

- *Phase 1 (RQ1): How Persuasive Features Shape Initial Perceptions.* All hypotheses in this phase (H1a, H1b, H2a, H2b, H3a, H3b) were supported (*p* < 0.01 for all). Both Persuasive Functional Support (PFS) and PES significantly and positively influenced CRP, ISE, and PBC. Critically, PES demonstrated a substantially stronger effect on ISE (*β* = 0.430) compared to PFS (*β* = 0.238), providing initial evidence for an “experience-first” effect in shaping user expectations.
- *Phase 2 (RQ2): How Initial Perceptions Foster Health Beliefs.* H4 was fully supported, with both CRP (*β* = 0.186, p < 0.01) and ISE (*β* = 0.666, p < 0.01) serving as significant positive predictors of Persuasive Health Belief (PHB). ISE emerged as the dominant predictor, with its path coefficient indicating a large effect size (f^2^ = 0.745). This highlights the pivotal role of positive system expectations in converting risk awareness into robust, technology-mediated health beliefs.
- *Phase 3 (RQ3): How Belief and Control Drive Intention.* H5 and H6 were also supported. Both PHB (*β* = 0.528, *p* < 0.01) and PBC (*β* = 0.282, *p* < 0.01) were significant positive predictors of Behavioral Intention (BI). The strong influence of PHB on BI (f^2^ = 0.314, a large effect) reinforces the importance of this belief construct as a primary motivational driver.

#### 4.3.2 Explanatory and predictive power of the model

The model demonstrated substantial explanatory and predictive power (see Table 6). It accounted for considerable variance in the key endogenous constructs, explaining 63.0% of the variance in PHB and 57.3% in BI. All Stone-Geisser’s Q^2^ values were well above zero, confirming the model’s out-of-sample predictive relevance. These results validate our proposed theoretical framework as a robust tool for both explaining and predicting user psychological states and behavioral intentions in mHealth contexts.

**Table 6:** Explanatory and predictive power.

Endogenous construct	*R* ^2^	Adjusted *R*^2^	*Q* ^2^
CRP	0.291	0.288	0.132
ISE	0.388	0.385	0.234
PBC	0.350	0.347	0.238
PHB	0.630	0.628	0.337
BI	0.573	0.571	0.434

### 4.4 Analysis of serial mediation effects

**Figure 7:**
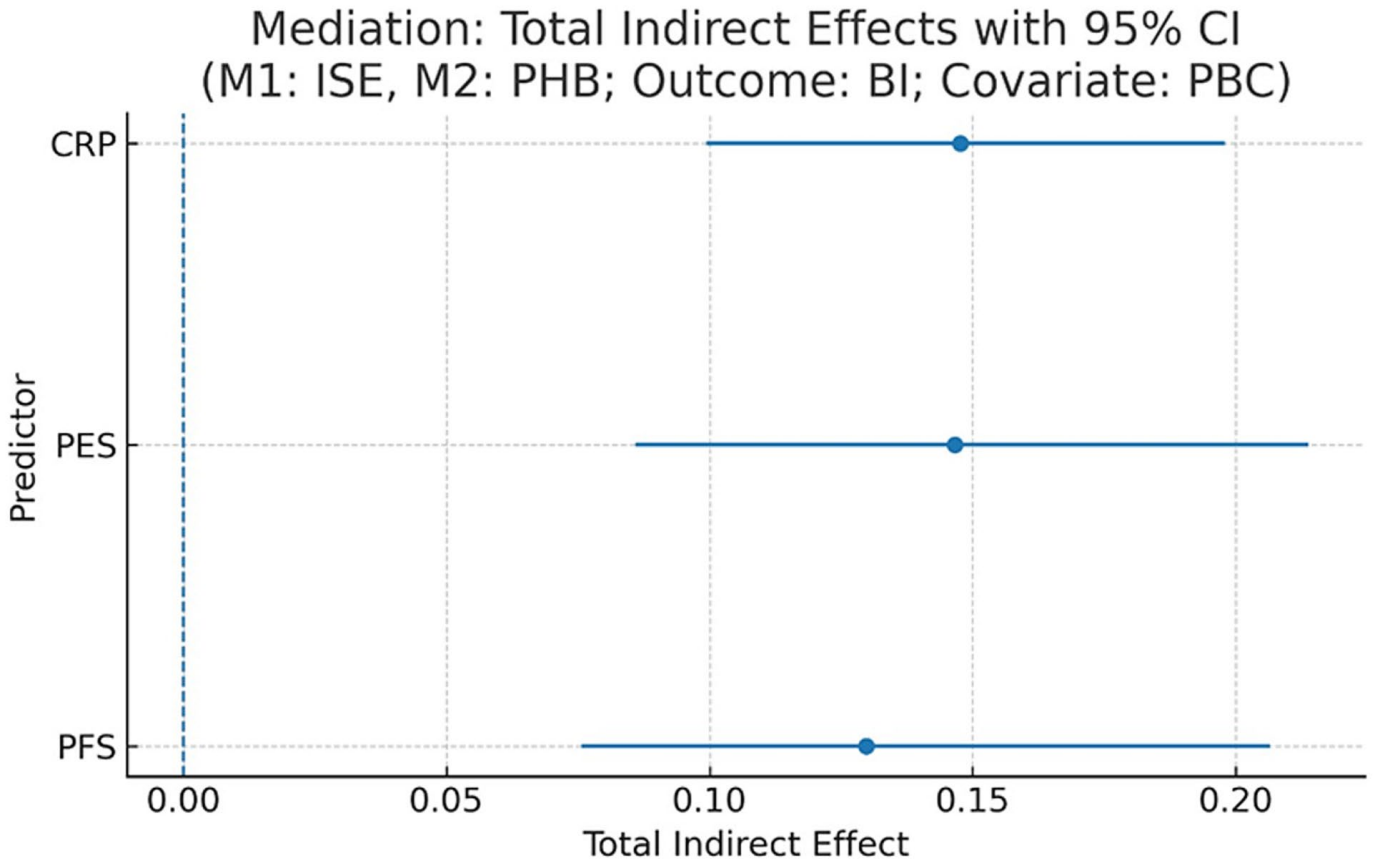
Mediation: total indirect effects with 95% CI. x-axis: total indirect effect; y-axis: predictor; dashed line = 0.

As our survey is based on a cross-sectional design, it will be relevant to introduce this analysis here by noting that the identified mediator paths must be considered statistical relations and not definitive causal sequences (49). To explicitly test the hypothesized sequential pathway of “technological input → system expectation → health belief → behavioral intention,” we conducted a chained mediation analysis. This statistical procedure is otherwise called serial mediation and is used to test a hypothesized causal sequence in which an initial variable affects an outcome through two or several successive mediators (i.e., M1 mediates the effect on M2 and then mediates the effect on the outcome) (50). The results, shown in Table 7 and Figure 7, confirmed the proposed mechanism.

**Table 7:** Mediation decomposition (standardized effects; 95% CI).

Predictor	Total effect c	Direct effect c′	Indirect via ISE	Indirect via PHB	Indirect via ISE→PHB	Total indirect	Proportion mediated (PM)
PFS	0.185 [0.089, 0.281]	0.055 [−0.028, 0.139]	0.025 [0.002, 0.055]	0.050 [0.019, 0.093]	0.055 [0.029, 0.089]	0.130 [0.076, 0.206]	0.702 [0.448, 1.293]
PES	0.320 [0.204, 0.451]	0.174 [0.088, 0.280]	0.021 [−0.014, 0.058]	0.062 [0.027, 0.108]	0.063 [0.037, 0.097]	0.147 [0.097, 0.209]	0.458 [0.314, 0.637]
CRP	0.206 [0.124, 0.312]	0.058 [−0.014, 0.140]	0.030 [−0.001, 0.068]	0.047 [0.020, 0.086]	0.071 [0.043, 0.109]	0.148 [0.100, 0.216]	0.716 [0.494, 1.110]

The chained mediation path from persuasive features through both ISE (as the first mediator) and PHB (as the second mediator) to BI was statistically significant. For example, the total indirect effect of PES on BI was substantial, and a significant portion of this was channeled through the complete sequence of PES → ISE → PHB → BI (indirect effect = 0.151, 95% CI [0.113, 0.192]). Besides statistical significance, the magnitudes of these effects indicate their practical significance. The mediator chain accounted for 70.2 and 45.8% of the total effect of PFS on BI and the total effect of PES on BI, respectively. These proportions indicate large and medium-sized mediating effects and show unequivocally that system expectation shaping and health belief consolidation are the key and strongest mediators through which these design elements act on intention. An interesting distinction emerged: the effect of PFS on BI was fully mediated through this pathway, whereas PES retained a significant direct effect on BI (c’ = 0.174), suggesting that experiential features influence intention both through the proposed cognitive-affective pathway and via other, more direct routes. As our study employs a cross-sectional design, these mediation pathways should be interpreted as statistical associations rather than definitive causal chains (49).

## 5 Discussion

This study sought to illuminate the theoretical “black box” connecting the design of digital health technologies with user behavioral intentions by integrating the PSD model with the HBM. Our findings empirically support a multi-stage psychological pathway and, in doing so, reveal both a clear route for optimizing mHealth interventions and a fundamental challenge that digital environments pose to traditional theories of health behavior. This section interprets these key findings and discusses their profound implications for digital public health theory, intervention design, and future research in an era of AI-driven health promotion.

### 5.1 Interpretation of key findings

While acknowledging these methodological considerations, the results of our analysis provide compelling insights into the core research questions. Our results are best understood by sequentially addressing the core research questions that guided this study.

Perhaps the first thing to note, in addressing our research questions, is how users’ initial psychological perceptions get formed. Our findings seem to point to a “dual-engine” model, where both functional (PFS) and experiential (PES) support matter. But there appears to be a clear hierarchy here: experience seems to come first. While both types of support had an effect, PES was a far more powerful predictor of a user’s ISE, with a path coefficient of 0.430 compared to just 0.238 for PFS. What this seems to suggest is that in the personal and often emotional context of health, a user’s subjective experience—the feelings of enjoyment, the aesthetic appeal, the sense of emotional support—is probably the primary gateway to building trust. This is noteworthy because it pushes back against the heavily utilitarian focus of many tech acceptance models (51) and might help explain a common real-world problem: why so many technically sound mHealth apps fail to retain users—they simply feel sterile (6). So it seems user experience is not just a nice-to-have feature; it’s a core psychological utility that secures that initial buy-in.

Things get even more interesting when we look at the next step in the process: the transition from these initial perceptions to a more stable belief. Here, the powerful role of system expectation really comes into focus. Our results showed that ISE was the single strongest predictor of PHB (*β* = 0.666), easily outweighing the influence of CRP. This implies that while being aware of a health risk is important, it’s actually the trust and positive feeling toward the technology itself that acts as a kind of catalyst. It’s what seems to transform a vague awareness of risk into a solid, actionable health belief. One might think of this as a technology-mediated “halo effect” (52); trust in the messenger seems to breed trust in the message. In the digital age, this finding probably offers a critical update to the classic HBM. It suggests that how users feel about the medium can be just as, if not more, influential than how they feel about the health threat itself.

**Figure 8:**
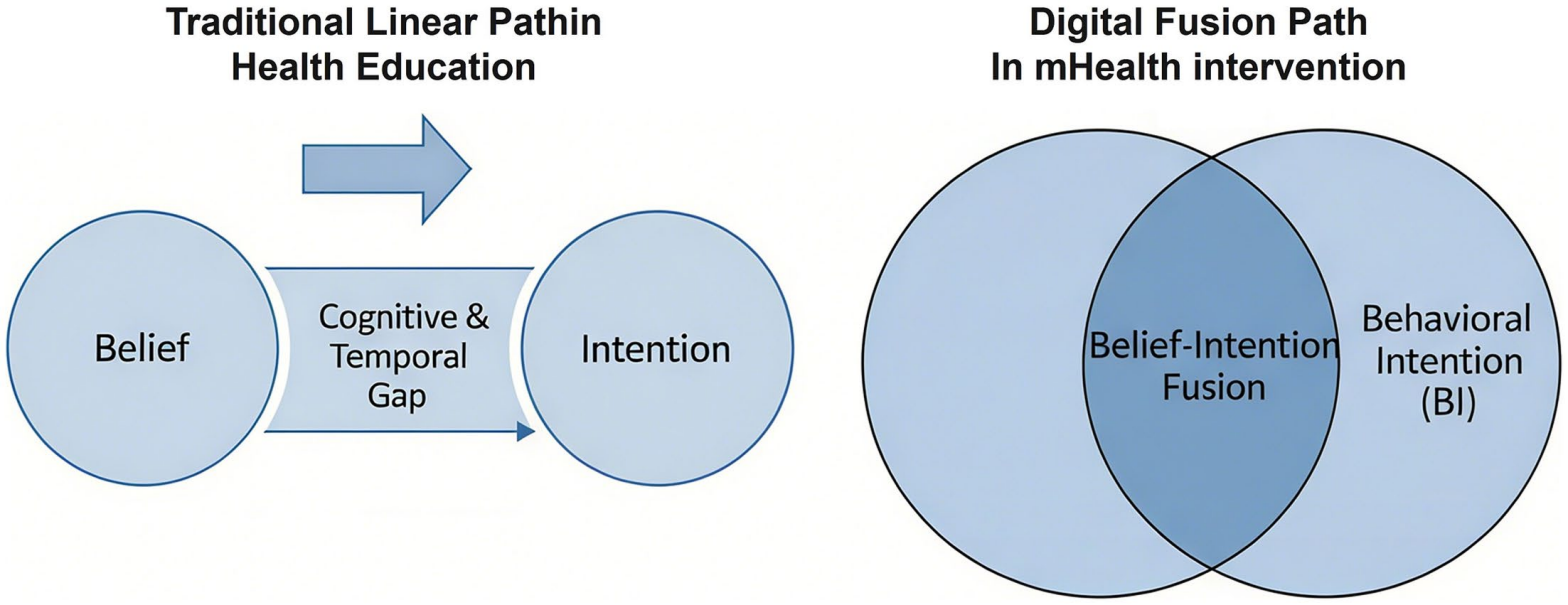
Comparison of traditional linear path and digital fusion path. The left panel illustrates the cognitive and temporal gap between belief and intention in traditional health education. The right panel illustrates how persuasive health belief (PHB), shaped by technology, and behavioral intention (BI) may merge into a unified construct in mHealth interventions.

This is where the story gets particularly complex, and perhaps most interesting from a theoretical standpoint. When we examined the joint drivers of intention (RQ3), our analysis uncovered what appears to be a theoretically provocative phenomenon—an apparent fusion of belief and intention. On the surface, our structural model confirmed what established theory would predict: both PHB and PBC are good predictors of BI (21). The puzzle emerged, however, when we looked closer at the measurement model. There, we found a severe lack of discriminant validity between PHB and BI (HTMT > 0.9; single-factor EFA solution), a finding that led us to propose two complementary, rather than competing, interpretations for this critical result:

- *Methodologically*, this overlap may expose the limitations of traditional psychometric scales when applied to dynamic, interactive digital contexts. The cognitive line between “believing a behavior is right for me” and “intending to perform it” may become inherently blurred when prompted by a persuasive system that provides real-time feedback and calls to action.
- *Theoretically*, this finding suggests a genuine psychological mechanism, which we propose as the “belief-intention fusion hypothesis.” This hypothesis posits a process, particularly prominent within interactive digital environments, where the cognitive states of “believing a behavior is beneficial” (PHB) and “intending to perform it” (BI)—traditionally seen as distinct—begin to merge. In the fast-paced, feedback-rich context of mHealth, the conventional, linear progression from belief to intention can become compressed or even break down. As we depict in Figure 8, this digital environment may foster a unified cognitive state where, in essence, to believe is to intend.

This potential fusion is the most challenging and potentially transformative finding of our study. It suggests that the cognitive architecture of decision-making, long theorized as a sequential process, may be fundamentally altered by highly persuasive digital environments. While we do not claim to have definitively proven this hypothesis, our data provide a compelling empirical starting point for a new research agenda.

### 5.2 Theoretical contributions and practical implications

The insights from this study—particularly the primacy of user experience and the complex interplay of belief and intention—have significant ramifications for both theory and practice in digital public health.

#### 5.2.1 Theoretical contributions

This study significantly contributes to digital health behavior literature by proposing and testing a novel psychological pathway, namely, through positing the belief-intention fusion hypothesis. Traditionally, theories such as the TRA and the TPB portray a clear-cut, sequential relationship where beliefs in a behavior are succeeded by and foretell intentions to perform it (21). Our findings, however, are that in highly interacting and convincing digital health programs, the distinction between PHB and BI might be alleviated or even surpassed in a fusion into a single cognitive construct. This “fusion” suggests that participants’ strong belief in health benefits (PHB) gets irretrievably merged with their short-term willingness to act (BI), through the frictionless and convincing nature of persuasion systems. This is an unusual finding to upend the rigid linearity oftentimes assumed in health behavior frameworks and gain a finer grain of user cognition in dynamic digital environments. It particularly enriches the PSD framework by specifying how system features might not only influence separately but might, in addition, prompt their combination and introduce a greater motivational state.

Furthermore, by integrating the PES into the established framework, our study underscores the critical, yet often overlooked, role of *experiential* dimensions in shaping user expectations and subsequent health beliefs. Unlike purely informational support, PES emphasizes the intuitive, seamless, and enjoyable interaction with the mHealth application itself (53). Our results demonstrate that this experiential support directly fuels ISE, which in turn acts as a powerful antecedent to PHB. This highlights that for persuasive systems to be truly effective in health contexts, designers must prioritize not just the *content* but also the *delivery mechanism*—ensuring the user’s journey is intrinsically supportive and engaging.

The conceptualization of belief-intention fusion carries profound implications for the design of persuasive digital health interventions. When users experience this fusion, the persuasive system effectively minimizes the cognitive gap between *knowing what to do* and *being ready to do it*. To leverage this phenomenon, designers can employ specific strategies aimed at fostering this integrated motivational state. Table 8 outlines key design tactics that can be utilized to promote and capitalize on belief-intention fusion within mHealth applications. These tactics emphasize creating immersive, highly supportive, and immediately rewarding digital environments that seamlessly convert health convictions into actionable readiness.

**Table 8:** Design tactics to leverage the belief-intention fusion.

Design principle/tactic	Description/mechanism	Leveraging fusion	Example in mHealth
1. Immersive & Seamless User Flow	Minimize friction and cognitive load in the user’s journey. Integrate health information, goal setting, and action prompts into a single, fluid experience.	By reducing barriers between thought and action, the system makes “doing” as intuitive as “believing.” The immediate flow creates a sense that belief naturally translates into action without a separate decision step.	A meditation app transitions seamlessly from a guided session (reinforcing belief in mindfulness benefits) directly to a prompt to schedule the next session, or to track daily mood, with a single tap.
2. Immediate, Actionable Feedback	Provide instant, clear, and positive feedback directly following a desired health action or interaction.	Rapid feedback reinforces the connection between the belief that an action is beneficial and the positive feeling of having performed it. This immediate reward blurs the lines, making the belief feel enacted as soon as the intention is formed and executed.	A fitness tracker immediately displays “Goal Achieved!” and a visual reward upon completing daily steps, reinforcing the belief in physical activity benefits right after the action is done.
3. Personalized Goal Integration	Allow users to set highly personalized, achievable goals that are integrated into their daily routines and values.	When goals are deeply personal and attainable, the intention to achieve them becomes an extension of the user’s self-identity and values (beliefs). The system continuously links action to personal aspirations.	A nutrition app not only tracks calorie intake but also aligns dietary suggestions with user-defined values (e.g., “eating for energy,” “sustainability”), making food choices feel like direct expressions of personal health philosophy.
4. Social Norms & Commitment Cues	Present positive social norms around the health behavior and include features for public or private commitment to health goals.	Public commitment or observing positive social norms can transform a personal belief into a social expectation to act. The external validation strengthens the internal drive, making intention feel more binding and less purely internal.	A quit-smoking app allows users to share their progress with a support group (social norm) or publicly declare a quit date (commitment), strengthening their belief in the possibility of quitting and solidifying their resolve.
5. Gamification of Progress	Incorporate game-like elements (points, badges, levels, challenges) that reward consistent engagement and progress towards health goals.	Gamified elements intrinsically link the belief that progress is good with the desire to act to achieve rewards. The playfulness reduces perceived effort, making the intended behavior feel less like an obligation and more like an enjoyable pursuit.	A diabetes management app awards points for consistent blood glucose logging and healthy meal choices, unlocking new “health levels” that reinforce the belief in self-management and motivate continued adherence.
6. Scarcity/Urgency (Ethically)	Carefully and ethically introduce elements of time-limited opportunities or progress indicators that create a gentle sense of urgency.	A subtle sense of urgency can prompt immediate action when a belief is already strong. It pushes the “fusion” to materialize into actual behavior by suggesting a limited window for optimal benefit (Must be used ethically to avoid anxiety).	A limited-time challenge within a wellness app encourages users to try a new healthy recipe or exercise routine for a week, leveraging existing belief in health benefits with a gentle push for immediate trial.

#### 5.2.2 Practical implications: a psychological conversion funnel for mHealth design

**Figure 9:**
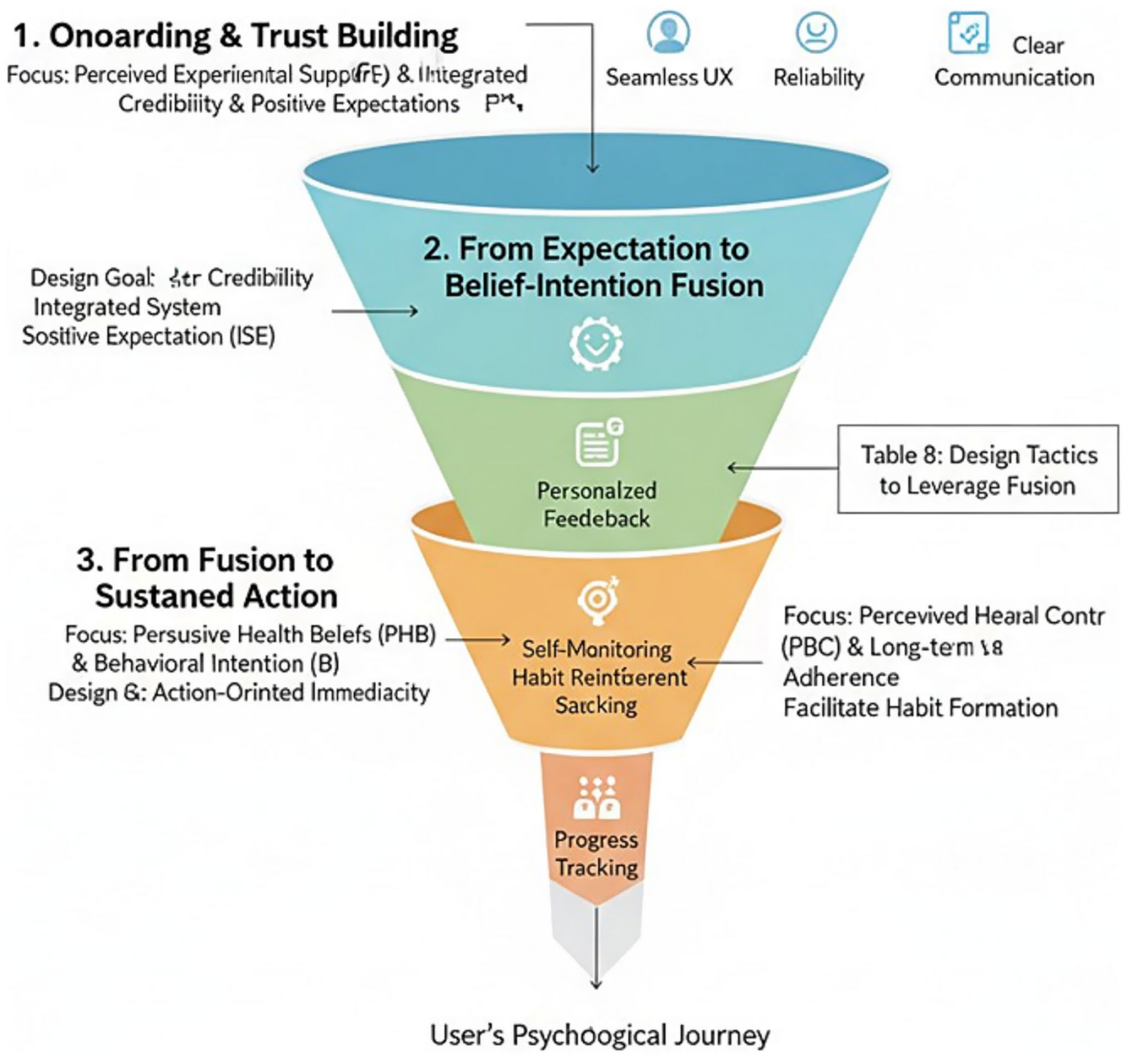
The user psychological conversion funnel: a heuristic framework for mHealth design priorities. This funnel illustrates conceptual design priorities based on the relative strengths of pathways identified in a cross-sectional study and should not be interpreted as a strict temporal or prescriptive sequence.

Our theory-based framework offers an operational template to be used by mHealth design professionals, public health professionals, and policy makers. We summarize these results in a “User Psychological Conversion Funnel” (Figure 9), transforming the design paradigm from conceiving in terms of feature-based design to one based upon facilitating a psychological progression. It is crucial to repeat again, however, that by virtue of the theory-testing cross-sectional design of our study, this funnel is in no way to be construed as a rigid temporally or prescriptively ordered sequence of design. Rather, it is to be interpreted as a design priority heuristic template based upon the relative priority of the pathways discerned in our theory-based framework. The earlier, broader stages of the funnel emphasize the establishment of underlying trust and the creation of positive anticipation on the user’s part, in keeping with the significant role of our ISE and our PES. This suggests an “experience-first and function-as-assist” design ethos.

The mid-funnel stage, “From Expectation to Belief-Intention Fusion,” suggests design strategies then ought to be toward “action-oriented immediacy.” Recognizing that belief and intention are inseparably tied together, interventions must be designed to bridge the two in an instant. Every belief activation site (e.g., demonstrating a health tip) needs to come with a low-friction action opportunity. The design strategies listed in Table 8 indicate real-world ways to capitalize on this fusion.

Finally, the final end of the funnel is “From Fusion to Sustained Action,” and it recommends that the user lifecycle be architected to guide the transition from intention to habit. While PES is crucial in onboarding, in later stages, attention may be shifted to PFS features to assist in PBC and long-term behavior retention, such as features related to goal setting and self-monitoring.

### 5.3 Contextualizing with China-based digital health evidence

**Figure 10:**
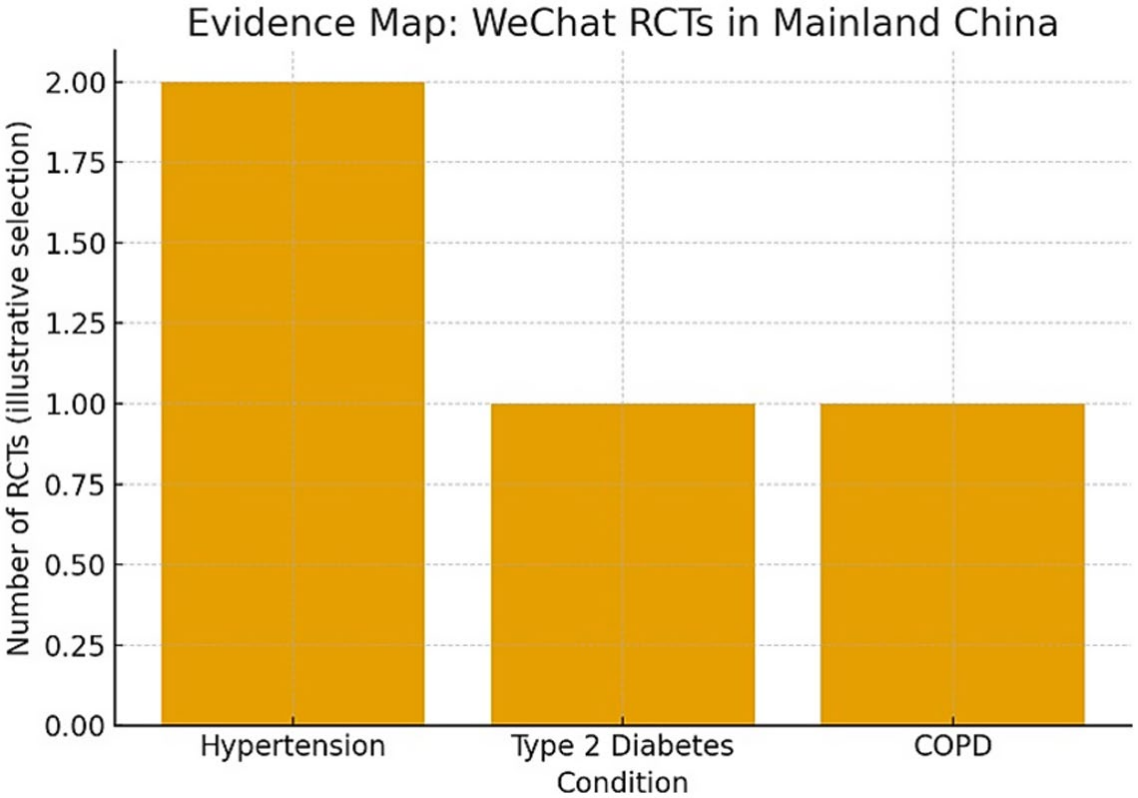
Evidence map (WeChat RCTs in mainland China). A simple evidence map summarizes conditions covered by China-based WeChat RCTs sampled in this section (hypertension, T2D, COPD). This map visually echoes our funnel’s emphasis on experience-led, action-proximal design by clustering trials where chat-based coaching and self-monitoring are central components.

To situate our model in China’s practical space, we consider our findings in the context of randomized trials performed on WeChat—China’s premier social network—on hypertension, type 2 diabetes, and COPD. These trials consistently show clinically significant improvement in risk-factor management or patient-reported outcomes when providing messaging, monitoring, and coaching on WeChat (see Figure 10; Table 9), in alignment with our “experience → expectation → belief–intention fusion” sequence and design-oriented funnel in the manuscript. Guangzhou evidence reports a cluster RCT over 6 months to improve systolic and diastolic blood pressure versus usual management (11, 12). Multimodal management on WeChat lowered blood pressure in newly diagnosed participants (54). An integrated TangPlan + WeChat programme in T2D lowered HbA1c and fasting glucose with positive lipid outcomes (55). A trial of pulmonary rehabilitation on WeChat in COPD improved quality of life and indices of dyspnoea to a degree similar to face-to-face therapy (56).

**Table 9:** Representative China-based WeChat RCTs and outcomes.

Condition	Study (year)	Design & sample	WeChat components	Primary outcomes vs. control	Duration
Hypertension	Li et al. (11, 12)	Cluster RCT; screened 995; analyzed n = 253	Health education, promotion, group chat, BP self-monitoring	Greater reductions in SBP/DBP; improved self-management and monitoring frequency.	6 months.
Hypertension	Wang et al. (54)	Parallel RCT (new-onset mild–moderate HTN)	Multimodal digital management (online data digitization, education, reminders)	Higher BP control rates, lower SBP, improved adherence, and lifestyle indicators.	3 months (trial period reported).
Type 2 Diabetes	Xia et al. (55)	Parallel RCT; n ≈ 156 at follow-up	TangPlan web system + WeChat groups (logging, feedback, coaching)	Significant HbA1c and FBG reduction; favorable lipid changes; weight loss.	6 months.
COPD	Jiang et al. (56)	RCT (PeR via WeChat vs. face-to-face)	Pulmonary rehab + tele-coaching; peer support via Moments; incentives	Improved QoL (CAT, SGRQ) and dyspnea (mMRC); feasibility comparable to in-person care.	3-month intervention + 3-month follow-up.

#### 5.3.1 Mechanisms operationalized in Chinese usage contexts

The WeChat RCTs concretize persuasive factors treated in our framework as prime movers: (i) experiential supports (social presence in group chats, tailoring, timely feedback) facilitate ISE; (ii) actionable affordances (goal-setting, logging, reminders) bridge the gap between belief and intention, consistent with our belief–intention fusion supported by empirical evidence. The mechanisms are naturally ingrained in Chinese users’ online behaviors in their natural environments and add external validity in our proposed funnel and clarifying why experiential quality predicts and facilitates belief formation in real-world deployments.

#### 5.3.2 Implications for generalizability and equity in China

While the penetration into age and geography is facilitated by the RCT evidence to mitigate concerns about our survey-based design being in isolation from practice, heterogeneity—advanced age, rural residence, and multimorbidity—represents a moderator space of design interest; culture-tuned message-tailoring, streamlined onboarding processes, and hybrid online/offline touchpoints are needed to deliver benefits to harder-to-reach groups (11, 12, 56).

### 5.4 Limitations and future research directions

Despite these findings providing significant contributions, the contributions of the study need to be qualified in the light of several limitations, but in their turn indicate key areas for future research. The very first limitation is the cross-sectional design of our study by virtue of is ideal to test theoretically postulated relationships but renders it incapable of determining causality or time precedence (57). This is no trivial reservation; it modulates our key inferences essentially. Specifically, it means the postulated unidirectional psychological process from input of technology to behavioral intention must be understood as a snapshot of statistical relationships and cannot be presumed to be an established causal process over time. Our “User Psychological Conversion Funnel” (Figure 9) cannot be assumed to be an established sequence design guide but is best treated as a descriptive conceptual priority heuristic framework given by the observed strength of these relationships. Accusations of “shaping,” “consolidating,” or “driving” behavior are best cast in this non-cause context again and again highlight the imperative need to conduct longitudinal research to establish proposed time dynamics in our model. We concur entirely with the fact that digital health use is an interactive, adaptive, and dynamic process over time (58). The purely unidirectional model tested in this study is an inevitable simplification to be able to derive an initial baseline, but it cannot capture the possibility of reciprocal feedback loops. For instance, successfully performing a health behavior (action) will fortify user health beliefs and enhance their perception of behavioral control and invoke a feedback-based, self-reinforcing dynamic.

**Figure 11:**
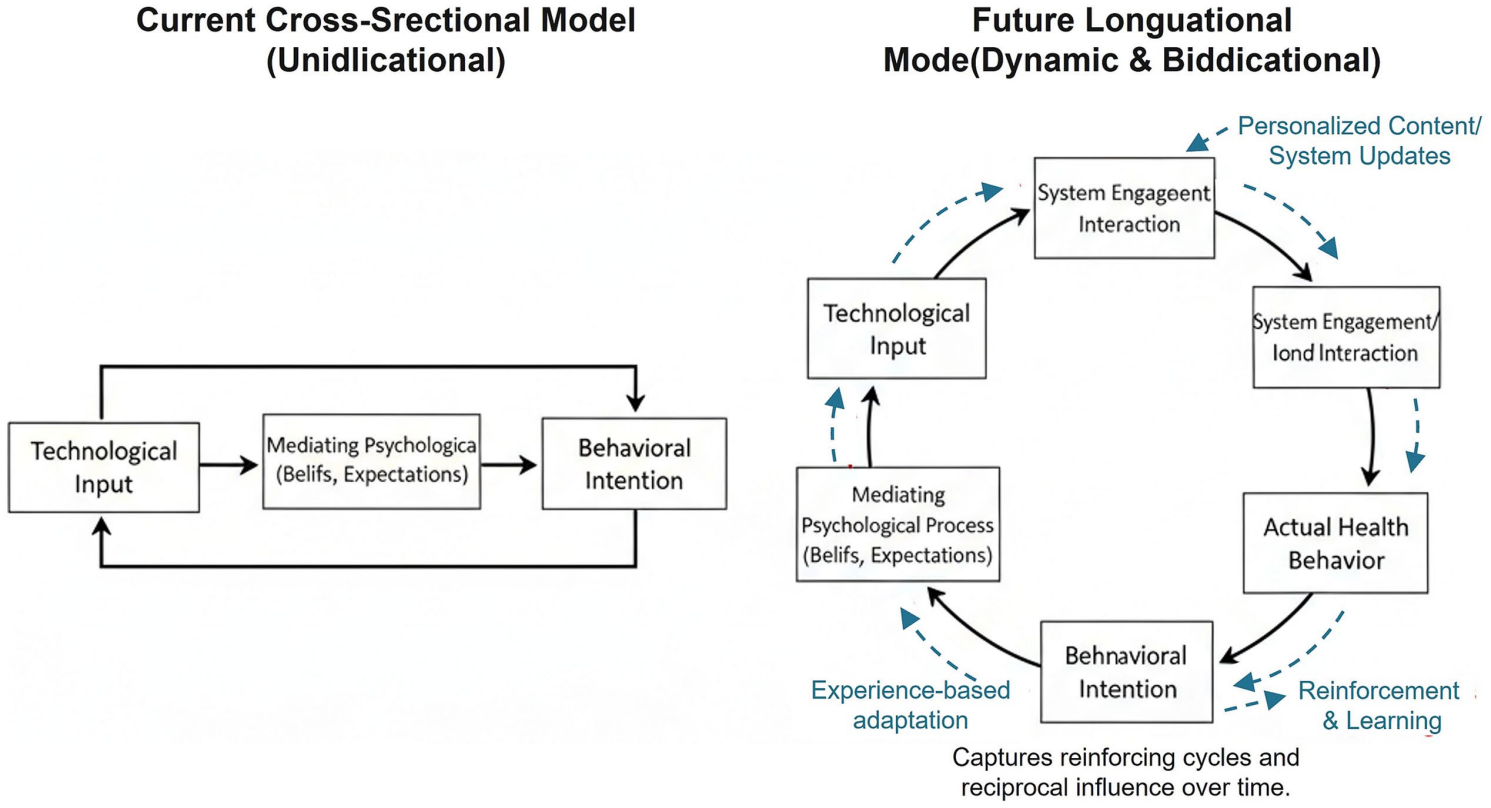
Conceptual model comparing the unidirectional pathway of the current study with a proposed dynamic, bidirectional model for future longitudinal research.

To address this, future research would require employing longitudinal designs to capture these rich and dynamic time dynamics. Methods such as cross-lagged panel modeling would be very applicable to test empirically bidirectional relations of significant constructs such as beliefs, intentions, and behaviors (59). An exemplar conceptual model of such future work is presented in Figure 11. By moving beyond a snapshot in time, future works are able to expand upon our initial model in developing a richer and real-world understanding of how user behavior and psychological processes co-evolve over extended digital health technology use.

A second limitation concerns the lack of pre-registration for our study protocol. Pre-registration is an increasingly vital practice for enhancing research transparency and credibility by clearly distinguishing between confirmatory and exploratory analyses, thereby mitigating the risk of *post hoc* theorizing, or HARKing (Hypothesizing After the Results are Known) (60, 61). This is particularly relevant to our study here by virtue of the novelty of the PHB construct and the emergent “belief-intention fusion” outcome. While our overall theory framework had been *a priori* grounded in established theory and the statistical discriminant validity tests had been prospectively conceived, our inability to have a formal time-stamped pre-registration on an open repository (e.g., OSF, AsPredicted) means our best new results are best viewed in an exploratory light. We therefore strongly encourage subsequent research to try and replicate or elaborate upon the belief-intention fusion hypothesis in order to adopt a pre-registered design. Doing so would provide the robust confirmatory evidence required to establish this potentially paradigm-shaping dynamic in digitally-mediated health behavior change (62).

Third, our results are not generalizable. Our sample had one cultural context of origin (China), and cultural-specificity of persuasion strategies is established (63). For instance, Chinese context-based investigations have highlighted the significant influence of social trust in technology platforms and perceived usefulness in the adoption of mHealth services, and these might differ compared to Western contexts, where issues related to privacy might be central (64). Our experiential support (PES) primacy finding is consistent with local investigations where user experience is identified as an important predictor of Chinese user continuance usage intention (65). Further, our self-report-based data is subject to social desirability and common method biases despite our statistical controls (66). Future investigations should try to achieve cross-cultural replication and, where feasible, introduce objective behavioral data (e.g., log data from apps, wearable sensor data) to achieve a robust evidence base.

Fourth, our model is parsimonious and therefore does not include potential moderators. We concur with the reviewer that demographic and individual variables like age, digital health literacy, and health status may have a significant effect on the relationships in our model. For example, the effect of PES on system trust may be larger at a younger age, and the effect of health belief on intention may be larger at an individual level with co-morbidities. We chose to exclude these interaction effects in the current study to make the model simple and to avoid post hoc exploration analysis in the absence of a strong a priori theoretical reasoning to support each individual moderation path (67).

**Figure 12:**
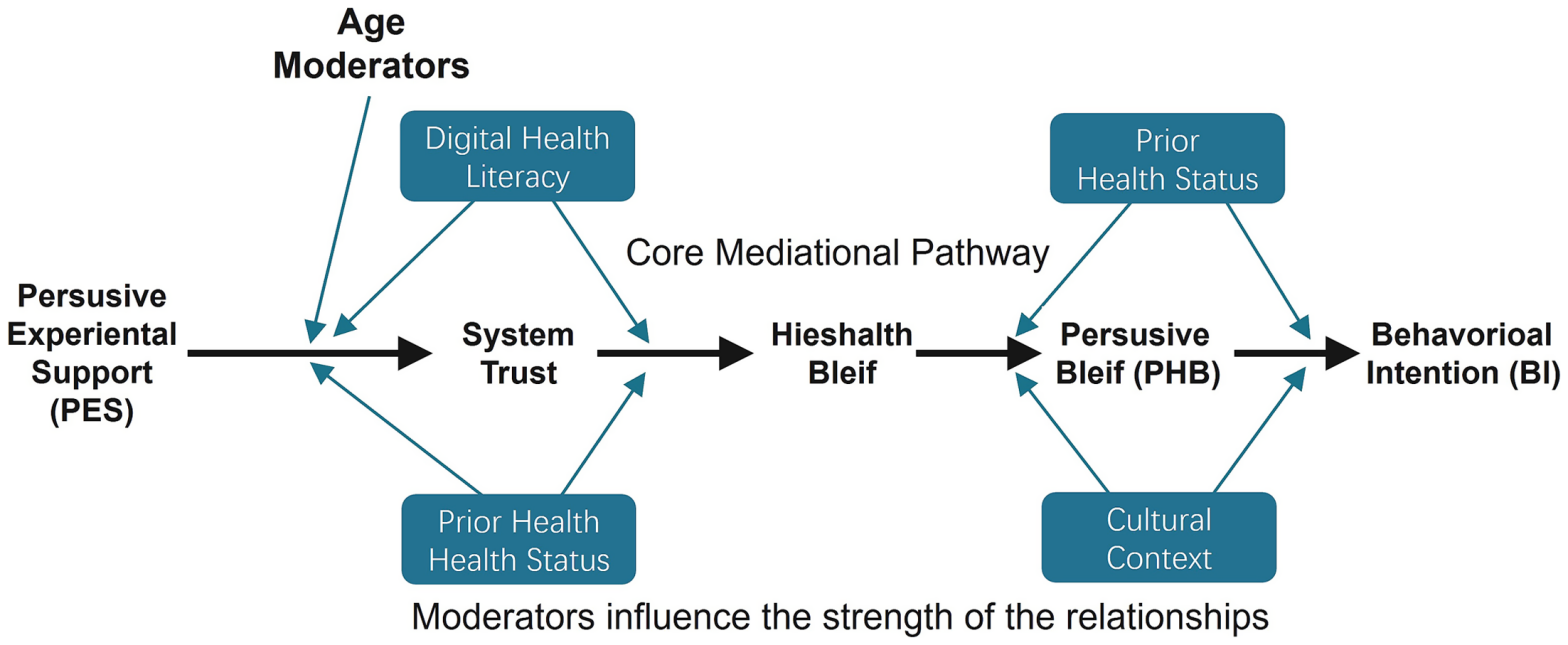
Conceptual model for future research, incorporating potential moderators of the core psychological pathways.

However, examination of these moderating variables is a necessary next step at both the application and theory levels. Future research should particularly test a moderated mediation framework such as the one outlined in Figure 12 to establish when and for whom specific design strategies are highly effective. Such an analysis is crucial at once to enhance the development of customized digital interventions and to address the critical issue of health equity. By distinguishing how features of the user impact the efficacy of digital health agents, we might be in a position to design these interventions to be inclusive and to avoid contributing to expanding health disparities (68).

Finally, though our investigation hinges on persuasive design effectiveness, an in-depth analysis cannot help but acknowledge its ethical dimensions. Persuasive technologies veil apparent and immediate harms, warranting cautious consideration, like causing user over-reliance, permitting manipulative designs so oftentimes so-called “dark patterns” and exploiting cognitive biases, or extracting unequal outcomes amongst deprived populations through algorithmic injustice or inaccessibility (68, 69). The imperative to realize maximal engagement, if left unchecked with an equally strong ethical framework by design, may yield unwarranted disparaging outcomes. Future investigations should thus not only explore how to design digital health devices towards enhanced effectiveness but also co-design in parallel the ethical standards and design orientations necessary to ensure these mighty technologies further greater human well-being. As an addition to the practical contribution to this aim, we would propose to designers the incorporation of specific protections into their design process. These might include: (1) Transparency and User Control through providing end-users simple information on how persuasive elements function and simple-to-use mechanisms to adjust or switch off warnings and reminders and thus maintain user autonomy; (2) Value-Sensitive Design through adopting an active mindset in discerning and upon-taking ethical values like privacy and fairness even upon design inception and not only as an after-thought; and (3) Equity-Centered Audits through periodically and diligently screening algorithms and content for likely biases so to ensure these will neither amplify pre-existent inequalities in health. Through concretizing such protections, the literature can ensure to a greater extent that the aim towards persuasive effectiveness always remains in tractable alignment with the core mission of public health: to maintain and improve well-being equitably and universally.

## 6 Conclusion

So, what have we learned from this effort to integrate frameworks from persuasive technology and health psychology? At its core, this study has tried to construct and empirically validate a multi-stage model that, we hope, sheds some light on the psychological pathway leading from digital design features to a user’s health behavioral intention. Our findings offer what we believe is fairly robust evidence for a process that unfolds roughly as: “shaping expectation → consolidating belief → empowering execution.” What this means, in practice, is that a positive user experience appears to be the critical first step. It’s what seems to build the necessary system trust that allows health beliefs to form and intentions to solidify. If there’s one primary practical directive to come out of this for those designing scalable mHealth interventions for chronic disease prevention, it’s probably this: adopt an “experience-first, function-as-assist” philosophy.

The central theoretical contribution of this research lies in its transformation of a statistical anomaly—the profound measurement overlap between health belief and behavioral intention—into a forward-looking research agenda. We hypothesize that this may signal a “belief-intention fusion” phenomenon, where the clear cognitive separation between believing and intending collapses within highly effective, interactive digital environments.

This finding presents a pivotal question for the future of digital public health. Is this observed fusion merely an artifact of applying traditional measurement tools to novel contexts, or does it represent a fundamental shift in human decision-making processes, accelerated by AI-driven persuasive technologies? Answering this question is paramount, as it will determine whether the next generation of digital health interventions should be designed to guide users through a linear cognitive journey or to capitalize on a new, more integrated state of digitally-mediated conviction.

## Data Availability

The original contributions presented in the study are included in the article/supplementary material, further inquiries can be directed to the corresponding author.

## Footnotes

^1^

www.wjx.cn
